# Fechamento Percutâneo de Canal Arterial Persistente em uma Gêmea Unida: Relato de Caso

**DOI:** 10.36660/abc.20240351

**Published:** 2024-12-11

**Authors:** Paulo Correia Calamita, Jonathan Guimarães Lombardi, Mayra Rosana Palmeira Barreto, Orlando Carlos Barbosa, Maurício Lopes Prudente, Zacarias Calil Hamu, Giulliano Gardenghi

**Affiliations:** 1 Hospital Estadual de Urgências Governador Otávio Lage Siqueira Goiânia GO Brasil Hospital Estadual de Urgências Governador Otávio Lage Siqueira (HUGOL), Goiânia, GO – Brasil; 2 Hospital Estadual da Mulher Goiânia GO Brasil Hospital Estadual da Mulher (HEMU), Goiânia, GO – Brasil; 3 Hospital ENCORE Goiânia GO Brasil Hospital ENCORE, Aparecida de Goiânia, GO – Brasil; 4 Clínica de Anestesia Goiânia GO Brasil Clínica de Anestesia (CLIANEST), Goiânia, GO – Brasil

**Keywords:** Gêmeos Unidos, Permeabilidade do Canal Arterial, Cateterismo

## Abstract

Gêmeos unidos com persistência do canal arterial e repercussões hemodinâmicas apresentam pior prognóstico. No presente relato de caso, demonstramos o primeiro fechamento percutâneo bem-sucedido do canal arterial com o dispositivo Piccolo© (Abbot Structural Heart, Plymouth, MN, EUA) nesse tipo de situação clínica.

## Introdução

A gestação múltipla ocorre em 1,6% de todas as gestações humanas. Considerando essa prevalência, 1,2% são dizigóticas e 0,4% são monozigóticas.^1,2^ Dessa pequena porcentagem de gêmeos monozigóticos, 5% são monocoriônicos e monoamnióticos e apenas 1% são gestações imperfeitas.^3^ A frequência de gêmeos fusionados (também chamados de gêmeos unidos) é estimada em cerca de 11 em 45.000 a 200.000 nascidos vivos,^1,4^ com predominância de 3:1 no sexo feminino.^1^

Os gêmeos unidos são classificados de acordo com o local de fusão mais proeminente: craniópagos (crânio), toracópagos (tórax), onfalópagos (abdômen), pigópagos (sacro), isquiópagos (pelve) e raquípagos (canal medular). Eles também podem ser divididos como simétricos (bem desenvolvidos) ou assimétricos (heterópagos), quando uma pequena parte do corpo é duplicada ou incompleta.^4^ Spencer^2^ também sugere que o lado da união seja aplicado para classificação: ventral (união no abdômen com um único umbigo) e dorsal (união no tubo neural, com o abdômen e o cordão umbilical separados). Grupo ventral rostral inclui o tipo cefalópago e o toracópago. O grupo caudal ventral inclui o isquiópago. O grupo ventrolateral inclui o parápago, e o grupo dorsal inclui o craniópago, raquípago e pigópago. Gêmeos parápagos são fetos com fusão ventrolateral. Eles podem ser unidos do abdômen inferior à pelve. Eles sempre têm uma única sínfise púbica e trato urinário.^5,6^

As malformações em gêmeos fusionados frequentemente são não concordantes.^7^ Elas incluem defeitos cardíacos congênitos, espinha bífida, higroma cístico, alterações nos membros, defeitos da parede abdominal, como gastrosquise e onfalocele, e hérnia diafragmática.^8^

No presente relato de caso, demonstramos um procedimento hemodinâmico realizado em uma gêmea unida com cardiopatia congênita, especificamente, persistência do canal arterial.

## Relato de Caso

**Figura 1:**
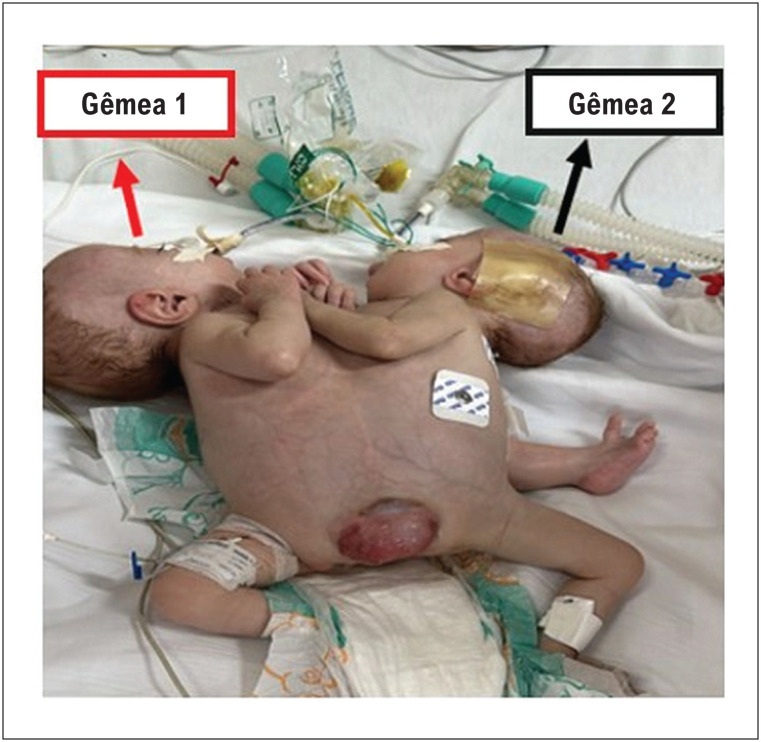
Gêmeas unidas fundidas ventralmente pelo tórax, apresentando abdome com assimetria dos membros inferiores.

**Figura 2:**
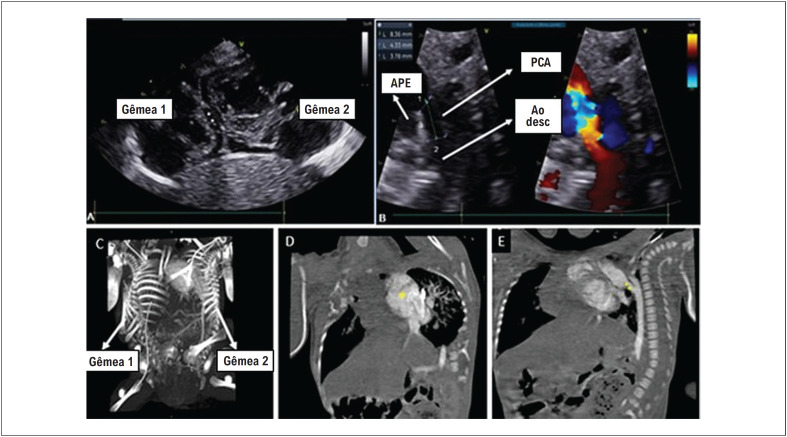
Ecocardiograma complementar e angiotomografia. A) Ecocardiograma mostrando 2 corações compartilhando o mesmo pericárdio. B) Corte ecocardiográfico identificando as seguintes estruturas: aorta descendente, artéria pulmonar esquerda e persistência do canal arterial e suas medidas, onde 1 = ampola pulmonar, 2 = ampola aórtica e 3 = comprimento. C) Tomografia com reconstrução óssea individualizando as gêmeas. D) * Coração da segunda gêmea. E) ** Persistência do canal arterial.

Relatamos o caso de gêmeas unidas prematuras (34 semanas e 3 dias) do sexo feminino, unidas ventralmente pelo tórax, abdome e pelve, com características heterópagas (assimetria: 3 membros inferiores: *tripus*), compartilhando o mesmo pericárdio (corações separados), fígado e bexiga, também sob investigação de fusão de outras estruturas abdominais (Figura 1) e com evidências de cardiopatia congênita na segunda gêmea. A persistência do canal arterial com repercussão hemodinâmica foi demonstrada por ecocardiograma (Figura 2A) e angiotomografia computadorizada (Figura 2C, 2D, 2E). Logo após o nascimento, a paciente desenvolveu hipoxemia, necessitando de intubação orotraqueal e ventilação mecânica com parâmetros ventilatórios elevados, evoluindo com hipertensão arterial persistente na recém-nascida, necessitando do uso de óxido nítrico, sildenafila e infusão contínua de milrinona.

As pacientes foram transferidas para um serviço de referência em cardiologia pediátrica aos 3 meses de idade, com parâmetros ventilatórios elevados (fração inspirada de oxigênio: 100%; pressão expiratória final positiva: 8 cmH_2_O; pressão inspiratória de pico: 24 cmH_2_O; frequência respiratória: 34 incursões respiratórias por minuto), difícil progressão alimentar e infusão contínua de milrinona. O fechamento medicamentoso do canal arterial com 3 doses de paracetamol enteral aos 15 dias de vida havia sido tentado anteriormente, sem sucesso. Optamos por não repetir o ciclo devido à dificuldade de utilização da via enteral e à ausência de paracetamol intravenoso no serviço. Estratégias terapêuticas foram discutidas com um *heart team*, optando-se pelo fechamento percutâneo do canal arterial. As pacientes foram transferidas para o laboratório de cateterismo, pesando 5,0 kg (estimado 2,5 kg para cada gêmea) para oclusão percutânea do canal arterial.

**Figura 3:**
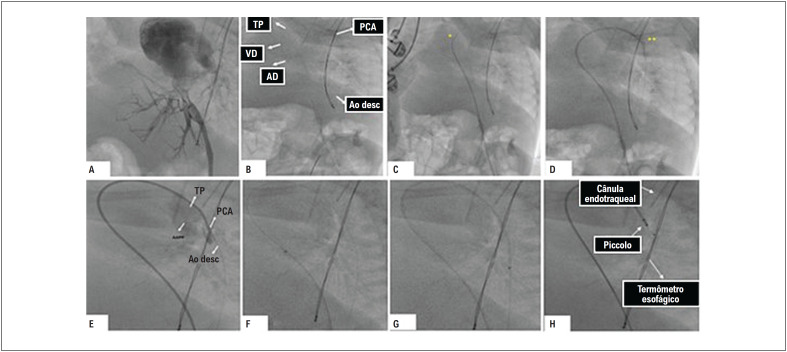
Passo a passo da intervenção percutânea. A) Angiografia no introdutor venoso mostrando a veia cava inferior drenando para o coração da segunda gêmea. B, C, D) Fluoroscopia mostrando o canal arterial cruzado com guia e cateter passando pelo átrio direito, ventrículo direito, tronco pulmonar na aorta descendente de forma anterógrada, e aorta e sendo posicionado na aorta descendente. E) Angiografia do canal arterial. F) Fluoroscopia mostrando o avanço da bainha do tronco pulmonar para a aorta descendente. G) Dispositivo Piccolo© e suas marcas radiopacas sendo posicionadas. O termômetro esofágico é geralmente um marco radiopaco para delimitar a ampola aórtica e a cânula orotraqueal é geralmente um marco radiopaco para delimitar a ampola pulmonar. AD: átrio direito; Ao: aorta; Ao Desc: aorta descendente; TP: tronco pulmonar; VD: ventrículo direito.

**Figura 4:**
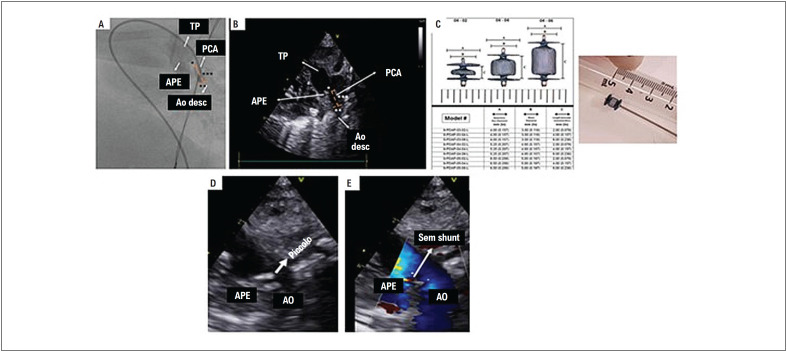
Correlação angiográfica (A) e ecocardiográfica (B) mostrando as medidas do canal arterial, sendo * ampola pulmonar, ** ampola aórtica e *** comprimento do canal arterial, fundamental para escolha do dispositivo. C) Dispositivo Piccolo©. D e E) Dispositivo liberado mostrando bom posicionamento final, sem shunt residual. Ao: aorta; Ao desc: aorta descendente; APE: artéria pulmonar esquerda; PCA: persistência do canal arterial; TP: tronco pulmonar.

Foi realizada a operação sob anestesia geral programada com 2 ventiladores mecânicos, 2 anestesiologistas e suprimentos dobrados. Foi administrada antibioticoterapia profilática durante a indução anestésica. A veia femoral do segundo membro inferior (pertencente à segunda gêmea) foi puncionada com auxílio de ultrassom e o introdutor transradial slender Terumo 4/5F foi posicionado. Foi realizada infusão em bolus de heparina 100 U/kg. Foi realizada angiografia no introdutor venoso para confirmar a drenagem venosa da veia cava inferior no coração da segunda gêmea (Figura 3A). Foi utilizado um cateter Judkins right 4F cordis em um guia 0,014" de 190 cm Balance Heavyweight para realizar angiografia no canal arterial, cruzado de forma anterógrada (Figura 3B), confirmando o diagnóstico de persistência do canal arterial. (Figura 3C). O guia 0,014" de 190 cm Balance Heavyweight foi posicionado na aorta descendente, abaixo do diafragma e sob o suporte da mesma bainha TorqVue 4F, avançado através do canal arterial (Figura 3D) até a aorta descendente para implantação do dispositivo, com base em medidas ecocardiográficas e angiográficas sequenciais (Figura 4A). Foi escolhido o Piccolo^©^ (Abbot Structural Heart, Plymouth, MN, EUA) 4,0 × 4,0 mm (Figura 4B). O cateter e o introdutor foram retirados, e o curativo oclusivo compressivo foi aplicado após a compressão hemostática manual.

Após o cateterismo, as pacientes evoluíram com estabilidade hemodinâmica. Retornaram ao hospital clínico pediátrico 24 horas após o cateterismo. A primeira gêmea foi extubada no sexto dia após o cateterismo, e a segunda gêmea no décimo segundo dia após o cateterismo.

## Discussão

No presente relato, demonstramos o primeiro fechamento percutâneo de canal arterial na segunda gêmea unida. Não encontramos relatos semelhantes em nossa revisão científica da literatura disponível sobre o tema. A oclusão do canal arterial garantiu melhora na insuficiência cardíaca congestiva na segunda gêmea, o que afetou diretamente o desenvolvimento da primeira gêmea. Dessa maneira, presume-se sucesso no desmame ventilatório e ganho de peso de ambas para desospitalização precoce e futura avaliação prognóstica quanto à probabilidade de cirurgia de separação.

A extensão da fusão e da anatomia intracardíaca é um dos fatores determinantes quanto ao potencial de separação e ao prognóstico a longo prazo em gêmeos unidos.^9^ A fusão cardíaca pode ser dividida em grupos de corações separados com pericárdio comum, átrios fusionados com ventrículos normais e átrios e ventrículos fusionados.^10-15^

No presente relato de caso, a fusão das gêmeas unidas foi ventral e se estendia entre as estruturas torácicas, abdominais e pélvicas, ocorrendo de forma assimétrica (3 membros inferiores com grau de deformação). Os corações eram separados, com um único pericárdio, e cardiopatia congênita também foi notada na segunda gêmea, a saber, persistência do canal arterial com repercussões hemodinâmicas.

Embora historicamente o uso de pelo menos 2 ciclos de anti-inflamatórios não esteroides seja o tratamento padrão ouro para oclusão do canal arterial no período neonatal, com taxa de sucesso de até 60% dos casos,^16^ as dificuldades de utilizar a via gástrica, as opções de anti-inflamatórios não esteroides disponíveis na instituição, bem como o potencial de eventos adversos não desprezíveis^16^ foram fatores limitantes para a terapia em questão. Foi tentado um ciclo de anti-inflamatórios não esteroides com paracetamol enteral, sem sucesso, no décimo dia de vida.

A escolha do cateterismo como opção para fechamento do canal arterial de gêmeos unidos, apesar da escassez de literatura para tratamento percutâneo em gêmeos unidos, foi devida à instabilidade clínica das gêmeas, ao potencial de eventos adversos diante de um novo ciclo de anti-inflamatórios não esteroides, aos riscos potenciais de instabilidade hemodinâmica imediatamente após a ligadura cirúrgica, potencialmente presente em até 45% dos casos,^16^ e aos excelentes resultados de ensaios clínicos de fechamento do canal arterial e com próteses dedicadas em pacientes prematuros com peso superior a 700 gramas a partir de 2020.^16,17^

O procedimento ocorreu sem intercorrências, com transporte e anestesia rigorosos. A via de acesso foi a punção venosa guiada por ultrassom do segundo membro inferior (após análise da angiotomografia) pertencente à segunda gêmea e foi guiada por ecocardiograma transtorácico tanto para mensuração do canal arterial quanto para posicionamento e liberação do dispositivo. O dispositivo foi bem posicionado dentro do canal arterial, sem shunt residual e sem obstrução ao fluxo das artérias pulmonar e aórtica esquerdas.

A evolução dos pacientes foi satisfatória, com extubação de ambos os pacientes e controle da insuficiência cardíaca.

## Conclusão

O presente relato de caso demonstra o primeiro fechamento percutâneo de canal arterial em uma paciente gêmea unido com uma prótese Piccolo^©^ (Abbot Structural Heart, Plymouth, MN, EUA) antes de planejar sua separação, com um resultado favorável, encorajando sua viabilidade.
