# Supplementary material for: Fechamento Percutâneo de Canal Arterial Persistente em uma Gêmea Unida: Relato de Caso
Source: Arq Bras Cardiol. 2024 Dec 11;121(12):e20240351. [Article in Portuguese] doi: 10.36660/abc.20240351 (PMC11634310; doi:10.36660/abc.20240351)
Supplement: Translation

# Translation: Percutaneous Patent Ductus Arteriosus Closure in a Conjoined Twin: Case Report

**DOI:** 10.36660/abc.20240351i

**Authors:** Paulo Correia Calamita, Jonathan Guimarães Lombardi, Mayra Rosana Palmeira Barreto, Orlando Carlos Barbosa, Maurício Lopes Prudente, Zacarias Calil Hamu, Giulliano Gardenghi

**Affiliations:** 1 Goiânia GO Brazil Hospital Estadual de Urgências Governador Otávio Lage Siqueira (HUGOL), Goiânia, GO – Brazil; 2 Goiânia GO Brazil Hospital Estadual da Mulher (HEMU), Goiânia, GO – Brazil; 3 Goiânia GO Brazil Hospital ENCORE, Aparecida de Goiânia, GO – Brazil; 4 Goiânia GO Brazil Clínica de Anestesia (CLIANEST), Goiânia, GO – Brazil

**Keywords:** Conjoined Twins, Patent Ductus Arteriosus, Catheterization

## Abstract

Conjoined twin patients with patent ductus arteriosus and hemodynamic repercussions have a worse prognosis. In the present case report, we demonstrate the first successful percutaneous closure of the ductus arteriosus with the Piccolo© device (Abbot Structural Heart, Plymouth, MN, USA) in this type of clinical situation.

## Introduction

Multiple gestation occurs in 1.6% of all human pregnancies. Considering this prevalence, 1.2% are dizygotic and 0.4% are monozygotic.^1,2^ Of this small percentage of monozygotic twins, 5% are monochorionic and monoamniotic, and only 1% are imperfect pregnancies.^3^ The frequency of fused twins (also called conjoined twins) is estimated to be around 1/45,000 to 200,000 live births,^1,4^ with a predominance of females 3:1.^1^

They are classified according to the most prominent fusion site: craniopagus (skull), thoracopagus (thorax), omphalopagus (abdomen), pygopagus (sacrum), ischiopagus (pelvis), and rachipagus (medullary canal). They can also be divided as symmetrical (well developed) or asymmetrical (heteropagus) unequal, when a small part of the body is duplicated or incomplete.^4^ Spencer^2^ also suggests that the side of the union be applied for classification: ventral (union at the abdomen with a single navel) and dorsal (union at the neural tube, with the abdomen and umbilical cord separated). The rostral ventral group includes the cephalopagus and thoracopagus. The ventral caudal group includes the ischiopagus. The lateral ventral group includes the parapagus, and the dorsal group includes the craniopagus, rachipagus and pygopagus. Parapagus twins are fetuses with ventrolateral fusion. They can be joined from the lower abdomen to the pelvis. They always have a single pubic symphysis and urinary tract.^5,6^

Malformations in fused twins are often non-concordant.^7^ They include congenital heart defects, spina bifida, cystic hygroma, limb changes, abdominal wall defects such as gastroschisis and omphalocele, and diaphragmatic hernia.^8^

In this case report, we demonstrate a hemodynamic procedure performed on a conjoined twin with congenital heart disease, specifically, patent ductus arteriosus.

## Case Report

We report the case of premature conjoined female twins (34 weeks and 3 days), joined ventrally by the thorax, abdomen, and pelvis, with heteropagus characteristics (asymmetry: 3 lower limbs: tripus), sharing the same pericardium (separate hearts), liver, and bladder, also under investigation into the fusion of other abdominal structures (Figure 1) and with evidence of congenital heart disease in the second twin. Patent ductus arteriosus with hemodynamic repercussions was demonstrated by echocardiogram (Figure 2A) and computed tomography angiography (Figure 2C, 2D, 2E). Shortly after birth, the patient developed hypoxemia requiring orotracheal intubation and mechanical ventilation with high ventilation parameters, progressing to persistent arterial hypertension in the newborn, requiring the use of nitric oxide, sildenafil, and continuous infusion of milrinone.

**Figure 1:** Conjoined twins fused ventrally through the thorax, showing abdomen with asymmetry of the lower limbs.

**Figure 2:** Complementary echocardiogram and tomography angiography. A) Echocardiogram showing 2 hearts sharing the same pericardium. B) Echocardiographic section identifying the following structures: descending aorta, left pulmonary artery, and persistence of the ductus arteriosus and its measurements, where 1 = pulmonary ampulla, 2 = aortic ampulla, and 3 = length. C) Tomography with bone reconstruction individualizing the twins. D) * Heart of the second twin. E) ** Patent ductus arteriosus. Desc Ao: descending aorta; LPA: left pulmonary artery; PDA: persistence of the ductus arteriosus.

The patients were transferred to a pediatric cardiology reference service at 3 months of age, with high ventilatory parameters (fraction of inspired oxygen: 100%; positive end-expiratory pressure: 8 cmH_2_O; peak inspiratory pressure: 24 cmH_2_O; respiratory rate 34 breaths per minute), difficult diet progression and continuous infusion of milrinone. Medical closure of the ductus arteriosus with 3 doses of enteral paracetamol at 15 days of life had been previously attempted, without success. We chose not to repeat the cycle due to the difficulty of using the enteral route and the absence of intravenous paracetamol in the service. Therapeutic strategies were discussed with a heart team, opting for percutaneous closure of the ductus arteriosus. The patients were transferred to the catheterization laboratory, weighing 5.0 kg (estimated 2.5 kg for each twin) for percutaneous occlusion of the ductus arteriosus.

The operation was performed under programmed general anesthesia with 2 mechanical ventilators, 2 anesthesiologists, and doubled supplies. Prophylactic antibiotic therapy was administered during anesthetic induction. The femoral vein of the second lower limb (belonging to the second twin) was punctured under the aid of ultrasound, and the transradial slender Terumo 4/5F introducer was positioned. Bolus infusion of heparin 100 U/kg was performed. Angiography was performed on the venous introducer to confirm the venous drainage of the inferior vena cava in the second twin's heart (Figure 3A). A Judkins right 4F cordis catheter was used on a 0.014" 190 cm Balance Heavyweight guidewire to perform angiography in the crossed ductus arteriosus in an anterograde manner (Figure 3B), confirming the diagnosis of patent ductus arteriosus. (Figure 3C). The 0.014" of 190 cm Balance Heavyweight guide was positioned in the descending aorta, below the diaphragm and under the support of the same TorqVue 4F sheath, advanced through the ductus arteriosus (Figure 3D) to the descending aorta for device implantation, based on sequential echocardiographic and angiographic measurements (Figure 4A). The Piccolo© (Abbot Structural Heart, Plymouth, MN, USA) 4.0 × 4.0 mm was chosen (Figure 4B). The catheter and introducer were removed, and compressive occlusive dressing was applied after manual hemostatic compression.

**Figure 3:** Step by step of the percutaneous intervention. A) Angiography in the venous introducer showing the inferior vena cava draining into the heart of the second twin. B, C, D) Fluoroscopy showing the ductus arteriosus crossed with guide and catheter passing through the right atrium, right ventricle, pulmonary trunk in the descending aorta in an anterograde fashion, and aorta and being positioned in the descending aorta. E) Angiography of the ductus arteriosus. F) Fluoroscopy showing the advancement of the sheath from the pulmonary trunk to the descending aorta. G) Piccolo^©^ device and its radiopaque marks being positioned. The esophageal thermometer is usually a radiopaque landmark to delimit the aortic ampulla, and the orotracheal cannula is usually a radiopaque landmark to delimit the pulmonary ampulla. Ao: aorta; Desc Ao: descending aorta; PT: pulmonary trunk; RA: right atrium; RV: right ventricle.

**Figure 4:** Angiographic (A) and echocardiographic (B) correlation showing the measurements of the ductus arteriosus, being * pulmonary ampulla, ** aortic ampulla, and *** length of the ductus arteriosus, fundamental for choosing the device. C) Piccolo© device. D and E) Device released showing good final positioning, without residual shunt. AO: aorta; Desc Ao: descending aorta; LPA: left pulmonary artery; PDA: persistence of the ductus arteriosus; PT: pulmonary trunk.

After catheterization, the patients developed hemodynamic stability. Twenty-four hours after catheterization, they returned to the pediatric clinical care hospital. The first twin was extubated on the sixth day after catheterization, and the second twin on the twelfth day after catheterization.

## Discussion

In this report, we demonstrate the first percutaneous closure of the ductus arteriosus in the second conjoined twin. We did not find similar reports in our scientific review of the available literature on the topic. The occlusion of the ductus arteriosus guaranteed improvement in congestive heart failure in the second twin, which directly affected the development of the first twin. In this way, success in ventilatory weaning and weight gain of both is assumed for early dehospitalization and future prognostic assessment regarding the probability of separation surgery.

The extent of fusion and intracardiac anatomy is one of the determining factors regarding separation potential and long-term prognosis in conjoined twins.^9^ Cardiac fusion can be divided into groups with separate hearts with common pericardium, atria fused with normal ventricles, and fused atria and ventricles.^10-15^

In the present case report, the fusion of the conjoined twins was ventral, and it extended between the thoracic, abdominal, and pelvic structures, occurring asymmetrically (3 lower limbs with a degree of deformation). The hearts were separate, with a single pericardium, and congenital heart disease was also noted in the second twin, namely, patent ductus arteriosus with hemodynamic repercussions.

Although historically the use of at least 2 cycles of non-steroidal anti-inflammatory drugs is the gold standard treatment for occlusion of the ductus arteriosus in the neonatal period, with a success rate of up to 60% of cases,^16^ the difficulties of using the gastric route, the available non-steroidal anti-inflammatory options at the institution, as well as the potential for non-negligible adverse events^16^ were limiting factors for the therapy in question. A cycle of non-steroidal anti-inflammatory drugs with enteral paracetamol was attempted, without success on the tenth day of life.

The choice of catheterization as an option for closing the ductus arteriosus of conjoined twins, despite the scarcity of literature for percutaneous treatment in conjoined twins, was due to the clinical instability of twins, the potential for adverse events in the face of a new non-steroidal anti-inflammatory drug cycle, the potential risks of hemodynamic instability immediately after surgical ligation, potentially present in up to 45% of cases,^16^ and the excellent results of clinical trials of ductus arteriosus closure with dedicated prostheses in premature patients weighing more than 700 grams from 2020.^16,17^

The procedure was uneventful, with strict transport and anesthesia. The access route was ultrasound-guided venipuncture of the second lower limb (after computed tomography angiography analysis) belonging to the second twin and was guided by transthoracic echocardiography both for measuring the ductus arteriosus and for positioning and releasing the device. The device was well positioned inside the ductus arteriosus, with no residual shunt and no obstruction to the flow of the left pulmonary and aortic arteries.

The patients’ evolution has been satisfactory, with extubation of both patients and control of heart failure.

## Conclusion

This case report demonstrates the first percutaneous occlusion of the ductus arteriosus in a conjoined twin patient with a Piccolo^©^ prosthesis (Abbot Structural Heart, Plymouth, MN, USA) before planning their separation, with a favorable result, encouraging feasibility.
